# Circulating microRNAs Correlated with Bone Loss Induced by 45 Days of Bed Rest

**DOI:** 10.3389/fphys.2017.00069

**Published:** 2017-02-14

**Authors:** Shukuan Ling, Guohui Zhong, Weijia Sun, Fengji Liang, Feng Wu, Hongxing Li, Yuheng Li, Dingsheng Zhao, Jinping Song, Xiaoyan Jin, Xiaorui Wu, Hailin Song, Qi Li, Yinghui Li, Shanguang Chen, Jianghui Xiong, Yingxian Li

**Affiliations:** ^1^State Key Laboratory of Space Medicine Fundamentals and Application, China Astronaut Research and Training CenterBeijing, China; ^2^Laboratory of Longitudinal Integration of Individual Life Data, Space Institute of Southern ChinaShenzhen, China; ^3^Key Laboratory of Molecular and Cellular Biology of Ministry of Education, College of Life Science, Hebei Normal UniversityShijiazhuang, China; ^4^National Key Laboratory of Human Factors Engineering, China Astronaut Research and Training CenterBeijing, China

**Keywords:** bed rest, bone loss, circulating miRNA, simulated microgravity, miR-1234

## Abstract

The purpose of this study was to find the circulating microRNAs (miRNAs) co-related with bone loss induced by bed rest, and testify whether the selected miRNAs could reflect the bone mineral status of human after bed-rest. We analyzed plasma miRNA levels of 16 subjects after 45 days of −6° head-down tilt bed rest, which is a reliable model for the simulation of microgravity. We characterize the circulating miRNA profile in individuals after bed rest and identify circulating miRNAs which can best reflect the level of bone loss induced by bed rest. Expression profiling of circulating miRNA revealed significant downregulation of 37 miRNAs and upregulation of 2 miRNAs, while only 11 of the downregulated miRNAs were further validated in a larger volunteer cohort using qPCR. We found that 10 of these 11 miRNAs (miR-103, 130a, 1234, 1290, 151-5p, 151-3p, 199a-3p, 20a, 363, and 451a) had ROC curve that distinguished the status after bed rest. Importantly, significant positive correlations were identified between bone loss parameters and several miRNAs, eventually miR-1234 showed clinical significance in detecting the bone loss of individuals after 45 days of bed rest.

## Introduction

Bone loss during long-term space flight is a key concern that poses significant risk to the health of astronauts (Wang et al., 2012). Bone mineral density (BMD) declines is caused by the imbalance of bone remodeling which is delicately regulated by both the number and activity of osteoblasts and osteoclasts (Wang et al., 2013; Zhao et al., 2015). It is estimated that the rate of bone mineral loss during spaceflight ranges from 0.5 to 1.5% per month, and its recovery requires three or four times longer time period (Sibonga, 2013). Until now, BMD can be measured by the “gold standard”- dual-energy X-ray absorptiometry (DXA). However, there is a challenge to obtain the small changes compared with the precision of the measurement (Wheater et al., 2013). The noninvasive, specific, and sensitive biomarker would be more feasible in this respect. Nevertheless, specific markers in individuals during spaceflight or mimic microgravity are not well-established. Only a few biomarkers concerning bone turnover exist including bone alkaline phosphatase (BALP), osteocalcin (OCN), and procollagen type I carboxy-terminal propeptide (PICP; Wang et al., 2012; Wheater et al., 2013). Head-down tilt (HDT) bed rest has proven to be a useful and reliable model for most physiological effects for spaceflight. This model has been used to test either resistive exercise or other countermeasures for prevention of microgravity-or disuse-induced bone loss (Wang et al., 2012; Yang et al., 2014).

Recent advances have identified miRNAs, which are noncoding, ~22-nucleotide-long RNAs that function as post-transcriptional regulators of gene expression, as key regulators of sophisticated gene expression to coordinate a broad spectrum of biological processes (Ling et al., 2013a,b; Zhao et al., 2015). Previous study by our group and others showed multiple miRNAs have been identified to regulate the complex process of bone remodeling (Wang et al., 2013; Chen et al., 2014). We found that miR-214 directly targeted ATF4 to inhibit osteoblast activity (Wang et al., 2013). Meantime, miR-214 promotes osteoclastogenesis by targeting Pten *in vitro* and *in vivo* (Zhao et al., 2015). miRNAs can be stable in plasma and serum and have also attracted interest as putative circulating biomarkers of diseases (Ward et al., 2014). Differential profiles of circulating miRNA have been reported for osteoporotic fractures (Seeliger et al., 2014), cardiovascular disease (McManus and Ambros, 2011; Roncarati et al., 2014), and cancer (Mitchell et al., 2008; Ortega et al., 2015). However, signatures of circulating miRNAs have not been characterized in individuals with bone loss induced by bed rest or mimic microgravity.

In this study, we demonstrate the miRNA pattern in the plasma of individuals after 45 days HDT bed rest. The levels of miRNAs that existed significant difference between pre- and post-bed rest were further assessed by quantitative real-time polymerase chain reaction in an independent cohort. The specificity and correlation of these miRNAs with bone loss index were analyzed by ROC curve and co-relation analysis, respectively. We identified miR-1234 correlated with BMD and bone biochemical indices of individuals after 45 days bed rest.

## Materials and methods

### Participants

Sixteen male volunteers were invited to participate in the 45 days of −6° HDT bed rest experiment. The volunteers were screened by a physical examination that included routine medical and laboratory tests to exclude chronic diseases-neurological disorders, musculoskeletal system disorders, infectious diseases and dyssomnia. Sixteen volunteers between 20 and 32 years old (mean = 26.6, *SD* = 4.2). And all volunteers had received greater than high school levels of education and were not taking medications, drugs, smoking, or alcohol.

A detailed explanation of the study (research purpose, whole experimental procedures and methods, conditions, possible problems, and complications) was provided, and written informed consent was subsequently obtained from each participant. Participants received financial compensation at the end of the study (Xu et al., 2013; Zhou et al., 2014). The study protocol conformed to the ethical guidelines of the 1975 Declaration of Helsinki. The study was approved by the Institutional Review Board of the China Astronaut Research and Training Center.

### Bone mineral density measurements

Areal bone mineral density (aBMD, in grams per square centimeter) of hip and lumbar spine (L2–L4) was measured by DXA (Osteocore-1, MEDILINK Company, France). The scans were performed according to standard clinical protocol for each site. In each scan, regions of interest (ROI) were defined by standard DXA protocol. Generally, BMD results were acquired by built-in software of DXA. Precision of DXA was calculated as the root mean squared coefficient of variation (%CV) for repeated measurements (Supplementary Table 2).

### Biochemical analysis

Biochemical analysis was performed according to manufacturer's protocol. Radioimmunoassay kits for measurement of serum osteocalcin (OCN) levels were purchased from Atomic Energy Institute of China (Beijing, China); bone alkaline phosphatase (BALP), procollagen type I C propeptide (PICP), and beta-carboxy-terminal cross-linking telopeptide of type I collagen (β-CTX) were measured by ELISA.

### Blood collection

Peripheral blood samples (2 ml) were collected into EDTA-containing tubes. Then whole blood was centrifuged at 1200 g for 15 min at room temperature within 30 min after blood collection, and the supernatant fluid was transferred into microcentrifuge tubes, followed by a second centrifugation at 12,000 g for 10 min at 4°C to remove cellular debris. Plasma was then aliquoted and stored at −80°C until use.

### MicroRNA microarray expression profiling

Total RNAs isolated from plasma of four individuals before and after 45 days of bed rest, respectively, were used for microRNA microarray profiling. Total RNA was extracted from 400 μl of plasma using the mirVanaTM RNA Isolation Kit (Applied Biosystems, Foster City, CA, USA) according to the manufacturer's specifications, and eluted with 100 μl of nuclease free water.

Total RNA (100 ng) was quantified by the NanoDrop ND-2000 (Thermo Scientific), and RNA integrity was assessed using Agilent Bioanalyzer 2100 (Agilent Technologies). miRNA microarray was performed and analyzed by the OEbiotech Corporation in Shanghai, P.R. China. Sample labeling, microarray hybridization and washing were performed as per the manufacturer's standard protocols. Briefly, total RNA were dephosphorylated, denatured and then labeled with Cyanine-3-CTP. After purification the labeled RNAs were hybridized onto the microarray (Release 19.0, Agilent) containing probes for 2006 human microRNAs. After washing, the arrays were scanned with the Agilent Scanner G2505C (Agilent Technologies) and the scanned images were analyzed using Agilent Feature Extraction Software (Agilent Technologies).

### Circulating miRNA extraction and detection by quantitative-PCR

miRNA from 200 μl plasma was extracted using the miRNeasy Serum/Plasma Kit, according to the manufacturer's recommendations (Qiagen, Germany). Briefly, samples were supplemented (after addition of QIAzol) with 3.5 μl miRNeasy Serum/Plasma Spike-In Control (1.6 × 10^8^ copies/μl working solution; Qiagen, Germany). The cel-miR-39 could be used for normalization of the RNA preparation. The amount and purity of RNA was estimated by quawell micro volume spectrophotometer (Quawell, USA). Subsequently, miRNA was transcribed to cDNA using the miScript II RT Kit (Qiagen, Germany). The cDNA were used for detecting miRNA expression by Q-PCR using the miScript SYBR Green PCR Kit with miScript Primer Assay (Qiagen, Germany). The relative expression level of miRNA was determined by the cycle number via Q-PCR, with levels normalized to the average of cel-miR-39 using the 2^−ΔΔCT^ method.

### Statistical analyses

Data are expressed as mean ± standard deviation (*SD*) or proportions where appropriate. Two-tail student's *t*-test was used to compare clinical characteristics between groups. MicroRNA data are presented as fold change relative to cel-miR-39 expression in each sample. Receiver operating characteristics (ROC) curves and the area under the ROC curve (AUC) were established to evaluate the diagnostic value of plasma microRNAs whose levels differed between before and post bed-rest. An AUC of 0.5 indicates classifications assigned by chance. Based on ROC analysis, the best statistical cutoff values of plasma microRNAs were calculated, and the sensitivity and specificity for selected cutoff points were then assessed. All statistical analysis was performed using Graphpad Prism 5.01 for Windows (Graphpad Software Inc., San Diego, CA, USA). Differences were considered statistically significant at a value of *P* < 0.05 (two-tailed).

## Results

### Participants characteristics

The microarray cohort of subjects included four individuals after 45 days of −6°HDT bed rest. For independent validation, in addition to the microarray cohort, we studied a second group composed of 16 individuals in this 45 days of −6°HDT bed rest experiment.

### Expression profiles of microRNAs in the serum of individuals after 45 days bed rest

**Figure 1:**
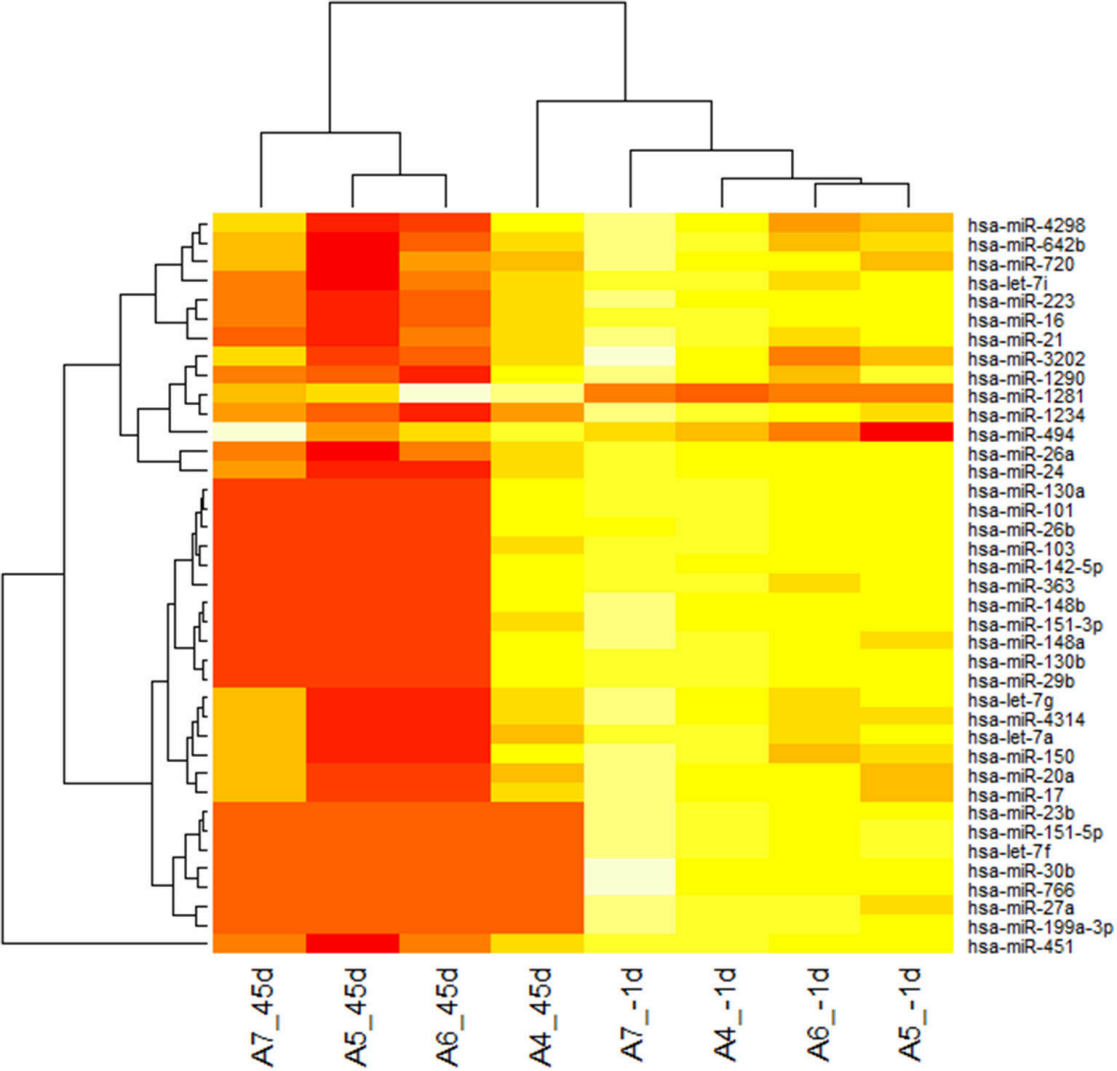
**Heat map of microRNA (miRNA) microarray expression data from plasma samples of individuals before and after 45 days of bed rest**. miRNA expression is hierarchically clustered on the y axis, and plasma samples from individuals before and bed rest are hierarchically clustered on the x-axis. The legend on the right indicates the miRNA represented in the corresponding row. The relative miRNA expression is depicted according to the color scale shown on the right. Red indicates downregulation; and yellow, upregulation. Numbers with A indicate the volunteer participated in the bed rest experiment, 45d indicated after 45 days of bed rest, −1d indicated 1 day before bed rest.

To determine the differential miRNA levels in individuals after 45 days of –6°HDT bed rest, we comparatively profiled serum miRNA expression of four individuals before and after the HDT bed rest, respectively (Baseline and BR-45). Paired sample *T*-test was used to analysis the microarray data. The levels of circulating miRNAs significantly differed after 45 days of bed rest, as illustrated in the heat map shown in Figure 1. Of 2006 miRNAs detected on the microarray, 39 miRNAs were found to be differentially expressed in individuals after 45 days bed rest (*P* < 0.05). Levels of 37 miRNAs were decreased and 2 miRNAs were increased in the plasma of individuals after 45 days bed rest. Fold changes of levels of miRNAs in the array are shown in Table 1.

**Table 1:** **Properties of microRNAs differentially expressed in plasma of individuals after 45 days of bed rest**.

**Downregulated miRNAs**	**Fold change**	***P*-value**	**Upregulated miRNAs**	**Fold change**	***P*-value**
hsa-miR-151-5p	0.19	0.000317	hsa-miR-1281	1.09	0.041138
hsa-let-7f	0.18	0.000467	hsa-miR-494	1.24	0.00532
hsa-miR-199a-3p	0.16	0.000519			
hsa-miR-23b	0.19	0.000875			
hsa-miR-30b	0.21	0.002515			
hsa-miR-27a	0.15	0.002705			
hsa-miR-766	0.25	0.004333			
hsa-miR-1234	0.95	0.00605			
hsa-miR-720	0.82	0.02101			
hsa-miR-17	0.43	0.022664			
hsa-miR-20a	0.43	0.023161			
hsa-miR-16	0.78	0.024081			
hsa-miR-21	0.78	0.026239			
hsa-let-7a	0.44	0.030984			
hsa-miR-103	0.3	0.031038			
hsa-let-7i	0.75	0.033246			
hsa-miR-151-3p	0.33	0.034416			
hsa-miR-26b	0.34	0.035724			
hsa-miR-642b	0.82	0.036784			
hsa-miR-451	0.84	0.037804			
hsa-miR-130a	0.33	0.038237			
hsa-miR-130b	0.39	0.03925			
hsa-let-7g	0.46	0.03935			
hsa-miR-3202	0.85	0.039886			
hsa-miR-363	0.37	0.040236			
hsa-miR-148a	0.36	0.040796			
hsa-miR-29b	0.38	0.040876			
hsa-miR-101	0.34	0.042404			
hsa-miR-1290	0.81	0.042588			
hsa-miR-142-5p	0.34	0.043007			
hsa-miR-223	0.77	0.045565			
hsa-miR-150	0.53	0.046573			
hsa-miR-24	0.42	0.04663			
hsa-miR-4314	0.5	0.047719			
hsa-miR-148b	0.36	0.047768			
hsa-miR-4298	0.88	0.047836			
hsa-miR-26a	0.48	0.049549			

### Quantitative reverse transcription polymerase chain reaction validation of the profiling data

**Figure 2:**
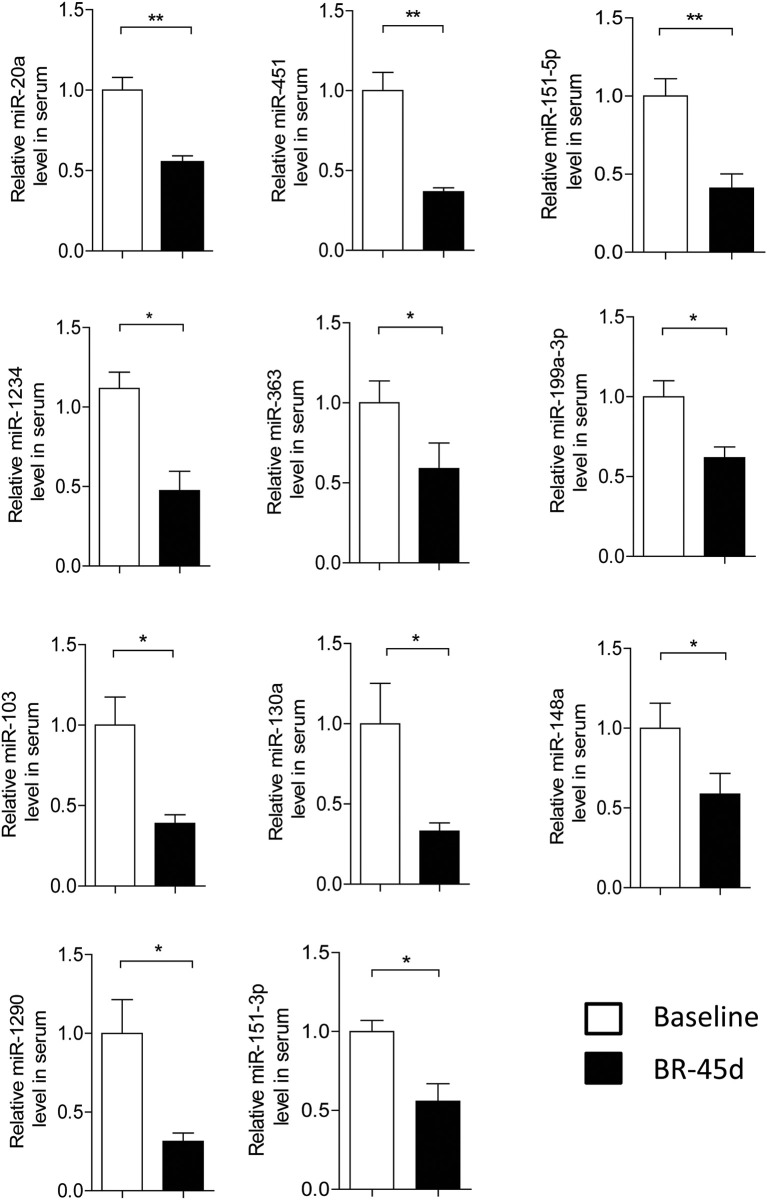
**Validation of microRNA (miRNA) microarray data by quantitative reverse-transcription polymerase chain reaction**. The relative levels of miRNAs were normalized to levels of the control (cel-miR-39). Baseline, pre-bed rest; BR-45d, post-45 days of bed rest. *n* = 4, the *P*-values were calculated by 2-tailed Student *t*-test. ^*^P < 0.05, ^**^P < 0.01.

To confirm miRNA profiling findings, we measured the levels of the 39 dysregulated miRNAs on the basis of their fold changes and *P*-values in microarray cohort via quantitative RT-PCR. The relative level of significantly changed miRNA in individuals is shown in Figure 2. Consistent with the profiling data, the levels of 11 miRNAs were decreased in individuals after 45 days bed rest (BR-45d) compared to baseline (Figure 2). Our results demonstrated miR-20a, 103, 451, 130a, 1234, 1290, 148a, 151-5p, 151-3p, 199a-3p, and 363 were significantly decreased and there were no significantly upregulated miRNAs in the plasma of individuals after 45 days bed rest.

### Independent validation of microRNA expression in individuals after 45 days of bed rest and 10 days of recovery

**Figure 3:**
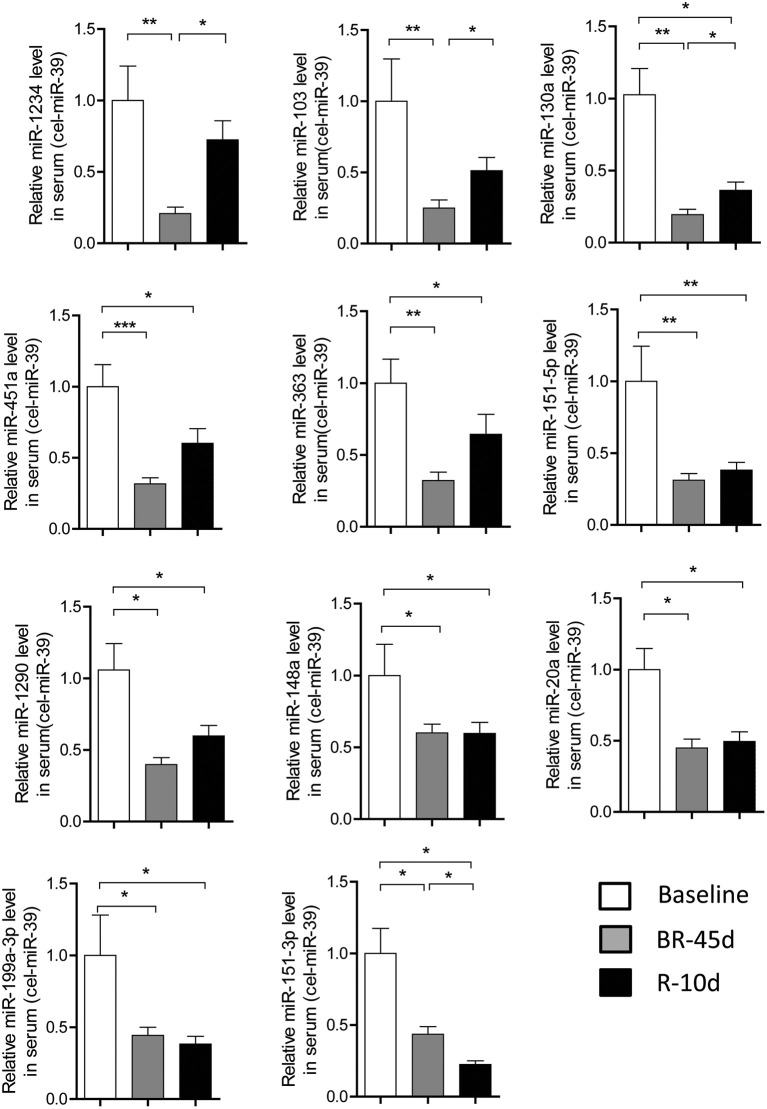
**Independent validation of microRNA expression in individuals after bed rest**. Quantitative reverse transcription polymerase chain reaction for 11 signature miRNAs in an independent validation set of 16 subjects. The relative levels of miRNAs were normalized to levels of the control (cel-miR-39). Baseline, pre-bed rest; BR-45d, post-45 days of bed rest; R-10d, recovery for 10 days. *P* values were calculated by 2-sided Student *t*-test. ^*^P < 0.05, ^**^P < 0.01, ^***^P < 0.001.

Next, we focused our studies on the 11 miRNAs: miR-103, 130a, 1234, 1290, 148a, 151-5p, 151-3p, 199a-3p, 20a, 363, and 451a which were verified by PCR on the basis of microarray results. To independently validate the levels of these circulating miRNAs, we studied a second set of plasma samples from 16 individuals after 45 days bed rest and 10 days of recovery. Figure 3 showed all these 11 miRNAs were significantly decreased in individuals after 45 days bed rest compared with the baseline values. In comparison with the BR-45d value, the levels of 3 miRNAs-miR-103, 130a, and 1234 were significantly higher in plasma of volunteers after 10 days of recovery. Interestingly, miR-151-3p level was on a continuous decline. These results suggested different circulating miRNA signatures might reflect the different physiological changes induced by HDT bed rest.

### ROC analysis for differential expression of circulating miRNAs

**Figure 4:**
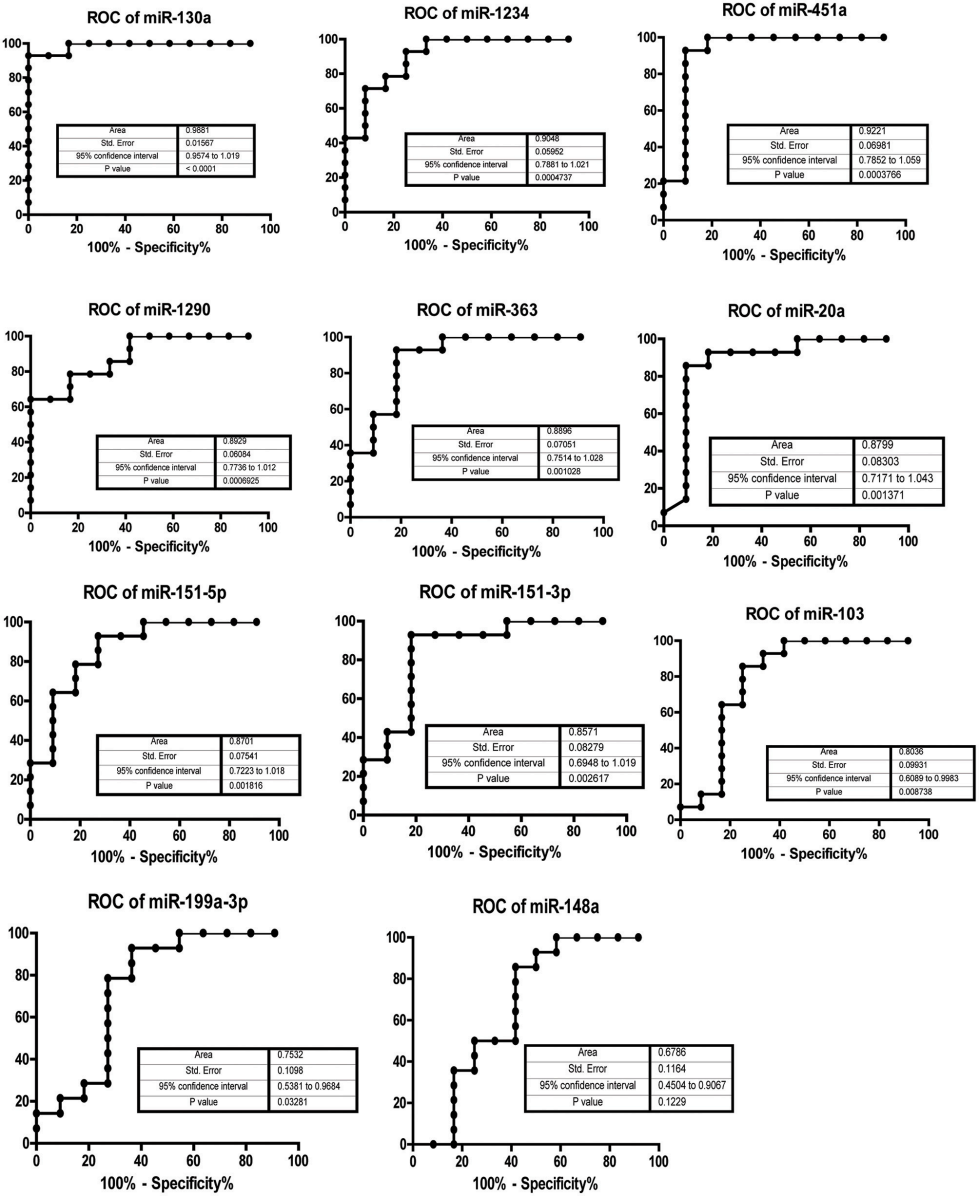
**AUC analysis of receiver-operating characteristics**. The area under the curve (AUC) (values given on the graphs) for miRNAs with significantly decreased circulating levels was calculated for physiological changes induced by 45 days of bed rest.

To assess the potential detective value of the significantly regulated miRNA in serum individuals after 45 days of bed rest, a ROC curve analysis was performed. The associated area under the curve (AUC) was used to confirm the diagnostic value of each miRNA. As shown in Figure 4, the AUC of miR-130a was the highest, reaching 0.9881 (95% confidence interval [CI] 0.9674–1.019, *p* < 0.0001). Besides miR-130a, miR-1234(AUC 0.9048, 95% CI 0.7881–1.021, *p* = 0.00047) and miR-451a (AUC 0.9221, 95% CI 0.7852–1.059, *p* = 0.00038) had an AUC > 0.90. The AUC of remaining eight miRNAs was 0.8929 for miR-1290 (95% CI 0.7736–1.012, *p* = 0.00069), 0.8896 for miR-363 (95% CI 0.7514–1.028, *p* = 0.0010), 0.8799 for miR-20a (95% CI 0.7171–1.043, *p* = 0.0014), 0.8701 for miR-151-5p (95% CI 0.7223–1.018, *p* = 0.0018), 0.8571 for miR-151-3p (95% CI 0.6948–1.019, *p* = 0.0026), 0.8036 for miR-103 (95% CI 0.6089–0.9983, *p* = 0.0087), 0.7532 for miR-199a-3p (95% CI 0.5381–0.9684, *p* = 0.033), and 0.6788 for miR-148a (95% CI 0.4504–0.9067, *p* = 0.12). The sensitivity and specificity associated with the optimal cut-off points are shown in Table 2. miR-130a showed the highest sensitivity of 92.6% and a specificity of 100%. The results suggested that these 10 miRNAs might be biomarkers of physiological changes induced by HDT bed rest.

**Table 2:** **Sensitivity and specificity of the regulated miRNAs in the serum of individuals after 45 days of HDT bed rest**.

**miRNAs**	**Sensitivity (%)**	**Specificity (%)**
miR-130a	92.9	100
miR-451a	92.9	90.9
miR-1234	92.9	75
miR-1290	64.3	100
miR-363	92.9	81.8
miR-20a	92.9	81.8
miR-151-5p	92.9	72.7
miR-151-3p	92.9	81.8
miR-103	85.7	75
miR-199a-3p	92.9	63.6

*Data were evaluated via a cut-off point*.

### Correlation of circulating miRNA level with bone loss induced by HDT bed rest

It is well known that bone loss during long-term space flight is a critical issue that poses significant risk to the health of astronauts. Bed rest has proved its usefulness as a reliable simulation model for bone loss during space flight (Wang et al., 2012). After we had determined the differential miRNA levels pattern, we examined whether any miRNA was correlated with the clinical prognostic variables of bone loss induced by HDT bed rest. In this bed rest experiment, all 16 subjects completed the 45 days bed rest. BMD at hip and lumbar spine (L2–L4) were lower after 45 days bed rest as compared with baseline. And after 10 days recovery, BMD at lumbar spine (L2–L4) were significantly increased as compared with BR-45d (Supplementary Figure 1). Bed rest had a significant effect on biochemistry markers—bone alkaline phosphatase (BALP), osteocalcin (OCN), procollagen type I carboxy-terminal propeptide (PICP) and beta-carboxy-terminal cross-linking telopeptide of type I collagen (β-CTX) (Supplementary Table 1). We use Pearson's correlation to analysis coefficients of significantly changed circulating miRNAs and bone parameters mentioned above. As shown in Table 3, the level of miR-1234 was significantly associated with hip BMD of subjects at total time points (*r* = 0.2853, *p* < 0.05) and 45 days of bed rest (*r* = 0.4581, *p* < 0.05), respectively. Table 4 showed the significant negative correlation between miR-20a and lumbar spine BMD at 45 days of bed rest (*r* = –0.5456, *p* < 0.05). However, no correlation with BMD was found for other eight miRNAs.

**Table 3:** **Pearson correlation coefficients of significantly decreased miRNAs and BMD at hip**.

	**Total**		**Baseline**		**BR-45d**		**R-10d**	
**Circulating miRNA**	**Corr with BMD**	***p***	**Corr with BMD**	***p***	**Corr with BMD**	***p***	**Corr with BMD**	***p***
miR-363	0.1008	0.5414	0.08712	0.7990	0.1745	0.5508	−0.08284	0.7783
miR-1290	−0.06863	0.6739	−0.3305	0.2941	−0.06903	0.8146	0.08109	0.7829
miR-103	0.1540	0.3427	0.06245	0.8471	0.03955	0.8932	0.3399	0.2344
miR-451a	0.2362	0.0739	0.1769	0.6029	0.04399	0.8813	0.3108	0.2795
miR-130a	−0.1561	0.3361	−0.4888	0.1068	−0.3902	0.1678	−0.1207	0.6810
miR-1234	**0.2853**	**<0.05**	0.2804	0.3773	**0.4581**	**<0.05**	0.1687	0.5816
miR-20a	0.1091	0.5086	0.02640	0.9386	0.1661	0.5705	−0.01745	0.9528
miR-199a-3p	0.1691	0.3035	0.1490	0.6619	0.1063	0.7176	0.2895	0.3153
miR-151-3p	0.1730	0.2991	0.1389	0.6837	0.3545	0.2136	0.03989	0.8970
miR-151-5p	0.1820	0.2675	0.1366	0.6888	0.3045	0.2899	0.1603	0.5841

*Values are Pearson correlation coefficient (r) and relative computed p value. Significant values are highlighted in bold. Total, sum of baseline, BR-45d and R-10d; Baseline, pre-bed rest; BR-45d, post-45 days of bed rest; R-10d, recovery for 10 days*.

**Table 4:** **Pearson correlation coefficients of significantly decreased miRNAs and BMD at Lumbar spine**.

	**Total**		**Baseline**		**BR-45d**		**R-10d**	
**Circulaing miRNA**	**Corr with BMD**	***p***	**Corr with BMD**	***p***	**Corr with BMD**	***p***	**Corr with BMD**	***p***
miR-363	−0.02957	0.8582	−0.03700	0.914	−0.4792	0.0830	−0.2003	0.4923
miR-1290	0.1550	0.3395	−0.1407	0.6627	0.3970	0.1599	−0.08242	0.7794
miR-103	−0.1342	0.4090	−0.4553	0.1369	−0.4420	0.1136	−0.03782	0.8979
miR-451a	0.1401	0.3949	0.05163	0.8802	−0.4132	0.1420	0.1450	0.6208
miR-130a	0.1187	0.4657	0.02214	0.9456	−0.4719	0.0885	−0.2118	0.4673
miR-1234	0.1305	0.4221	0.1886	0.4259	−0.2051	0.4819	−0.1577	0.5903
miR-20a	−0.02151	0.8966	−0.3191	0.3388	−**0.5456**	**<0.05**	0.4088	0.1467
miR-199a-3p	−0.1276	0.4390	−0.3479	0.2945	−0.4506	0.1059	0.09329	0.7511
miR-151-3p	0.03943	0.8142	−0.1416	0.6778	−0.1099	0.7083	−0.06473	0.8336
miR-151-5p	−0.03541	0.8305	−0.3249	0.3297	−0.3059	0.2875	0.2138	0.4630

*Values are Pearson correlation coefficient (r) and relative computed p value. Significant values are highlighted in bold*.

In addition, significant correlations were found for miR-1290 (*r* = −0.2706, *p* < 0.05), miR-130a (*r* = −0.2984, *p* < 0.05), and miR-1234 (*r* = −0.3545, *p* < 0.05) with total serum BALP of individuals at total time points respectively. Meantime, miR-130a (*r* = −0.5439, *p* < 0.05) and miR-1234 (*r* = −0.7367, *p* < 0.01) were significantly associated with serum BALP at 45 day of bed rest (Table 5). As shown in Table 6, miR-1234 had significantly positive correlation with serum OCN at 45 days of bed rest (*r* = 0.7288, *p* < 0.01). Table 7 showed serum PICP at total time points were significantly associated with miR-363 (*r* = −0.3347 *p* < 0.05), miR-1290 (*r* = −0.2908, *p* < 0.05), miR-103 (*r* = −0.2739, *p* < 0.05), miR-451a (*r* = −0.3204, *p* < 0.05), miR-130a (*r* = −0.3402, *p* < 0.05), miR-1234 (*r* = −0.2396, *p* < 0.05), and miR-20a (*r* = −0.3148, *p* < 0.05), respectively. At 45 days of bed rest, miR-451a (*r* = −0.5007, *p* < 0.05) and miR-1234 (*r* = 0.7418, *p* < 0.01) had significant positive correlations with serum PICP, and only miR-130a (*r* = −0.5859, *p* < 0.05) were significantly associated with serum PICP at 10 days of recovery. Table 8 showed no significant correlation with β-CTX which was a marker of bone resorption was found for all miRNAs. These results indicated that miR-1234 had significant correlations with most of bone parameters of individuals in this bed rest experiment. Circulating miR-1234 might be the potential biomarker of bone loss induced by HDT bed rest or spaceflight.

**Table 5:** **Pearson correlation coefficients of significantly decreased miRNAs and Serum BALP**.

	**Total**		**Baseline**		**BR-45d**		**R-10d**	
**Circulaing miRNA**	**Corr with BALP**	***p***	**Corr with BALP**	***p***	**Corr with BALP**	***p***	**Corr with BALP**	***p***
miR-363	−0.2427	0.0683	−0.2617	0.4370	−0.3038	0.2910	0.01739	0.9530
miR-1290	−**0.2706**	**<0.05**	−0.4842	0.1107	0.2535	0.3818	−0.2405	0.4075
miR-103	−0.1632	0.3144	−0.04615	0.8868	−0.2208	0.4482	−0.2770	0.3378
miR-451a	−0.2271	0.1644	−0.2162	0.5231	−0.3907	0.0836	0.08602	0.7700
miR-130a	−**0.2984**	**<0.05**	−0.3791	0.2243	−**0.5439**	**<0.05**	−0.3022	0.2937
miR-1234	−**0.3545**	**<0.05**	−0.2663	0.4029	−**0.7367**	**<0.01**	−0.08088	0.7834
miR-20a	−0.1033	0.5314	−0.07099	0.8357	−0.3149	0.2728	0.1044	0.7225
miR-199a-3p	−0.01692	0.9186	0.01166	0.9728	−0.3357	0.2406	0.09405	0.7491
miR-151-3p	0.06645	0.6918	0.01067	0.9752	−0.07889	0.7886	−0.05065	0.8695
miR-151-5p	−0.03009	0.8557	−0.002424	0.9944	−0.1267	0.6661	0.1696	0.5622

*Values are Pearson correlation coefficient (r) and relative computed p-value. Significant values are highlighted in bold. BALP, Bone alkaline phosphatase*.

**Table 6:** **Pearson correlation coefficients of significantly decreased miRNAs and Serum OCN**.

	**Total**		**Baseline**		**BR-45d**		**R-10d**	
**Circulaing miRNA**	**Corr with OCN**	***p***	**Corr with OCN**	***p***	**Corr with OCN**	***p***	**Corr with OCN**	***p***
miR-363	0.08861	0.5917	0.3045	0.3625	0.3550	0.2130	−0.02822	0.9237
miR-1290	0.1865	0.2491	0.2595	0.4155	0.05659	0.8476	0.4043	0.0758
miR-103	0.1241	0.4456	0.1696	0.2991	0.3863	0.1724	0.2024	0.4876
miR-451a	0.02562	0.8769	0.2699	0.4222	0.4168	0.1382	−0.1551	0.5966
miR-130a	0.07062	0.6650	0.2430	0.4467	0.1731	0.5541	0.1633	0.5769
miR-1234	0.1839	0.2560	0.3477	0.1330	**0.7288**	**<0.01**	0.1559	0.5945
miR-20a	−0.05131	0.7564	0.1746	0.6077	0.3747	0.1868	0.1559	0.5945
miR-199a-3p	−0.0003102	0.9985	0.1561	0.6467	0.3215	0.2623	0.03708	0.8998
miR-151-3p	−0.07235	0.6660	0.08965	0.7932	0.08592	0.7710	0.2542	0.4021
miR-151-5p	−0.01071	0.9484	0.1724	0.6121	0.1824	0.5557	−0.08378	0.7758

*Values are Pearson correlation coefficient (r) and relative computed p value. Significant values are highlighted in bold. OCN, osteocalcin*.

**Table 7:** **Pearson correlation coefficients of significantly decreased miRNAs and Serum PICP**.

	**Total**		**Baseline**		**BR-45d**		**R-10d**	
**Circulaing miRNA**	**Corr with PICP**	***p***	**Corr with PICP**	***p***	**Corr with PICP**	***p***	**Corr with PICP**	***p***
miR-363	**0.3347**	**<0.05**	0.3471	0.2956	0.4141	0.0705	0.2323	0.2121
miR-1290	**0.2908**	**<0.05**	0.3110	0.1626	0.1007	0.366	−0.1253	0.6695
miR-103	**0.2739**	**<0.05**	0.2005	0.5322	0.4337	0.0606	0.08559	0.7711
miR-451a	**0.3204**	**<0.05**	0.3073	0.3580	**0.5007**	**<0.05**	0.1034	0.7250
miR-130a	**0.3402**	**<0.05**	0.2640	0.4069	0.2323	0.4242	**0.5859**	**<0.05**
miR-1234	**0.2396**	**<0.05**	0.06216	0.8478	**0.7418**	**<0.01**	0.1092	0.7103
miR-20a	**0.3148**	**<0.05**	0.2013	0.5528	0.4376	0.0588	0.1243	0.6721
miR-199a-3p	0.2081	0.2036	0.1720	0.6130	0.3248	0.2572	−0.2362	0.4163
miR-151-3p	0.1751	0.2929	0.1271	0.7095	0.1059	0.7186	−0.4227	0.0751
miR-151-5p	0.2029	0.2154	0.1878	0.5804	0.1456	0.6195	−0.2113	0.4684

*Values are Pearson correlation coefficient (r) and relative computed p value. Significant values are highlighted in bold. PICP, procollagen type I carboxy-terminal propeptide*.

**Table 8:** **Pearson correlation coefficients of significantly decreased miRNAs and Serum β-CTX**.

	**Total**		**Baseline**		**BR-45d**		**R-10d**	
**Circulaing miRNA**	**Corr with β-CTX**	***p***	**Corr with β-CTX**	***p***	**Corr with β-CTX**	***p***	**Corr with β-CTX**	***p***
miR-363	−0.05057	0.7598	0.03450	0.9198	0.3273	0.1267	−0.08325	0.7772
miR-1290	−0.1554	0.3384	−0.3130	0.3219	−0.2736	0.3439	0.06888	0.8150
miR-103	−0.08435	0.6048	−0.07971	0.8055	0.06375	0.8286	−0.2439	0.4007
miR-451a	−0.1384	0.4008	−0.001824	0.9958	0.4477	0.1084	−0.3440	0.2285
miR-130a	−0.09952	0.5467	−0.2118	0.5087	0.2636	0.3624	−0.2589	0.3931
miR-1234	−0.1319	0.4172	−0.2859	0.3677	0.2087	0.2370	0.1067	0.7167
miR-20a	−0.06211	0.7072	0.2802	0.4039	0.2947	0.1532	−0.4969	0.0706
miR-199a-3p	0.08236	0.6182	0.3519	0.2886	0.3928	0.1648	−0.2017	0.4892
miR-151-3p	0.05143	0.7591	0.2571	0.4454	0.3620	0.2034	−0.01387	0.9641
miR-151-5p	0.003863	0.9814	0.3416	0.3039	0.1764	0.5463	−0.2807	0.3311

*Values are Pearson correlation coefficient (r) and relative computed p value. β-CTX, beta-carboxy-terminal cross-linking telopeptide of type I collagen*.

## Discussion

In this study, we report for the first time the association of certain serum circulating miRNAs with bone loss induced by 45 days of bed rest. We characterize the circulating miRNA profile in individuals after bed rest and identify circulating miRNAs which can best reflect the level of bone loss induced by bed rest. Expression profiling of circulating miRNA revealed significant downregulation of 37 miRNAs and upregulation of 2 miRNAs, while only 11 of the downregulated miRNAs were further validated in a larger volunteer cohort using qPCR. We found that 10 of these 11 miRNAs had an ROC curve that distinguished the status after bed rest. Importantly, significant positive correlations were identified between bone loss parameters and several miRNAs, eventually miR-1234 showed clinical significance in detecting the bone loss of individuals after 45 days of bed rest.

To our knowledge, the function of miR-1234 in bone remodeling remains unknown. Only a few studies showed miR-1234 might play a role in regulation of tumorigenesis of B-cell lymphoma (Hogfeldt et al., 2014) and used miR-1234 as biomarker for predicting survival in patients with nasopharyngeal carcinoma (Liu et al., 2014). Functional analysis in targetScan 6.0 showed that miR-1234 could target different genes involved in bone remodeling and in basic biological processes such as cell-cell signaling, DNA replication, cell death and survival. Some of differentially expressed circulating miRNAs appear to be involved in the biologic processes of bone remodeling. miR-103 inhibits osteoblast proliferation through suppression of Cav1.2 expression under simulated microgravity condition *in vitro* (Sun et al., 2015). And miR-20a can promote osteogenic differentiation of human mesenchymal stem cells by co-regulating BMP signaling (Zhang et al., 2011). miR-130a play an important role in regulating the expression of TNF-α in human chondrocytes (Li et al., 2015).

miR-451a, miR-363, miR-1290, miR-363, miR-151-5p, and miR-151-3p had an optimal area value under the curve >0.85, indicating that these circulating miRNAs levels might be useful biological markers for assessing the risk of bed rest or simulated microgravity. However, no correlation with BMD after 45 days of bed rest was found for these miRNAs. Bed rest has proved its usefulness as a reliable simulation model for most physiological effects of spaceflight, for example muscle wasting, cardiovascular deconditioning, adverse metabolic changes, and bone demineralization (Jost, 2008). Except bone loss, these miRNAs levels may have correlation with other physiological effect of bed rest-muscle wasting, cardiovascular deconditioning, or adverse metabolic changes.

miRNAs have been demonstrated to play crucial roles in many physiological and pathophysiological processes (Ling et al., 2013a,b,c; Wang et al., 2013; Zhao et al., 2015). To date, only a very few studies have demonstrated that specific miRNA involved in bone loss induced by spaceflight or simulated microgravity (Wang et al., 2013). Our previous study indicated that miR-214 directly targeted ATF4 to inhibit osteoblast activity and bone formation. And we found simulated microgravity (hindlimb unloading model)—induced deleterious changes in trabecular bone mass and trabecular architecture were efficiently attenuated by the inhibition of miR-214 in mice (Wang et al., 2013). Unfortunately, the microRNA microarray results showed no significant change in miR-214 level in this study, however qPCR results demonstrated circulating miR-214 levels were significantly increased in plasma of individuals after bed rest (unpublished data). Therefore, the significantly changed miRNA in plasma of individuals after bed rest may be involved in the bone loss induced by spaceflight or disuse.

To date, there have been several biochemical markers of bone turnover, for instance, BALP, OCN, PICP, and so on (Lee and Vasikaran, 2012; Wheater et al., 2013; Al Nofal et al., 2015). However, these biomarkers have different disadvantages. The serum BALP levels have up to 20% cross reactivity with liver isoforms (Seibel, 2005), OCN levels are unstable in serum and have short half-life of a few minutes (Lee and Vasikaran, 2012), and PICP also have short half-life and are sensitive to thyroid hormones and IGF-1(Szulc and Delmas, 2008). miRNAs are circulating freely in the mammalian blood and can be predicted as biomarker for early diagnosis of disease (Guay and Regazzi, 2013). Circulating miRNAs are stable under different storage conditions and can be measured using assays that are specific, sensitive, and reproducible (Seeliger et al., 2014). So far, expression profiling of circulating miRNAs as biomarkers revealed both diagnostic and prognostic potential in cardiovascular disease (McManus and Ambros, 2011) and more than 10 types of cancer (Mo et al., 2012). Also, circulating miRNAs can be used as biomarkers for diagnostic purpose in osteoporotic patients (Seeliger et al., 2014). However, signatures of circulating miRNAs have not been characterized in individuals with bone loss induced by disuse or bed rest. To our knowledge, this is the first study to evaluate the circulating miRNA fingerprint in bed rest people and analyze the correlation between circulating miRNA levels and bone loss induced by bed rest.

In conclusion, the present study provides the evidence of an altered circulating miRNA expression profile in HDT bed-rest people. Ten circulating miRNAs (miR-103, 130a, 1234, 1290, 151-5p, 151-3p, 199a-3p, 20a, 363, and 451a) have potential detective value for distinguishing the physiological changes after bed-rest, and miR-1234 might be the diagnostic biomarker of bone loss induced by bed rest or disuse.

## Author contributions

SL provided the concept and design of the study, analysis and interpretation of data, and drafting of the manuscript; GZ, WS, and FW performed the experiment; FL analyzed the data of microarray; HL, YuHL, DZ, and JS assisted with manuscript preparation; XJ, XW, HS, and QL collected the samples from subjects; YiHL, SC, JX, and YXL revised the manuscript and gave final approval of the submitted manuscript. All authors have read and approved the final manuscript.

### Conflict of interest statement

The authors declare that the research was conducted in the absence of any commercial or financial relationships that could be construed as a potential conflict of interest.
