# Supplementary material for: Circulating microRNAs Correlated with Bone Loss Induced by 45 Days of Bed Rest
Source: Front Physiol. 2017 Feb 14;8:69. doi: 10.3389/fphys.2017.00069 (PMC5306370; doi:10.3389/fphys.2017.00069)

# Circulating MicroRNAs Correlated with Bone Loss Induced by 45 days of Bed Rest

Shukuan Ling[1]†, Guohui Zhong[1]†, Weijia Sun[1]†, Fengji Liang[1,2]†, Feng Wu[1], Hongxing Li[1,3], Yuheng Li[1], Dingsheng Zhao[1], Jinping Song[1], Xiaoyan Jin[1], Xiaorui Wu[1], Hailin Song[1,3], Qi Li[1], Yinghui Li[1,2], Shanguang Chen[4] , Jianghui Xiong[1, 2]*, Yingxian Li[1]*

[1] State Key Laboratory of Space Medicine Fundamentals and Application, China Astronaut Research and Training Center, Beijing, China.

[2] Laboratory of Longitudinal Integration of Individual Life Data, Space Institute of Southern China, Shenzhen, China

[3] Key Laboratory of Molecular and Cellular Biology of Ministry of Education, College of Life Science, Hebei Normal University, Shijiazhuang, China.

[4] National Key Laboratory of Human Factors Engineering, China Astronaut Research and Training Center, Beijing, China

†These authors contributed equally to the work.

* Address for Correspondence:

Yingxian Li, Ph.D., State Key Lab of Space Medicine Fundamentals and Application, China Astronaut Research and Training Center, No. 26 Beiqing Road, Haidian District, Beijing, 100094, China. Tel: 8610-62895755, E-mail: yingxianli@aliyun.com

or Jianghui Xiong, Ph.D., State Key Lab of Space Medicine Fundamentals and Application, China Astronaut Research and Training Center, No. 26 Beiqing Road, Haidian District, Beijing, 100094, China., E-mail: xiongjh77@163.com

**Supplementary table 1**

**Bone formation and turnover markers before, during, and after 45 days of bed rest.**

| Biochemical indices | Baseline | BR-45d | R-10d |
|---|---|---|---|
| BALP (serum, ng/ml) | 27.16±8.67 | 33.54±10.51 ** | 23.93±5.12 |
| OCN (serum, ng/ml) | 9.91 ±2.19 | 8.47 ±2.71* | 13.91±9.40 # |
| PICP (serum, ng/ml) | 43.95±17.95 | 31.38±20.77 * | 37.37±10.54 |
| β-CTX (serum, ng/ml) | 0.66 ± 0.36 | 0.83 ± 0.49 | 0.73 ± 0.45 |

Data are expressed as mean ± SD of values, n=16, *$P$<0.05, ** $P$<0.01, compared with baseline; #$P$<0.05, compared with BR-45d. BALP: Bone alkaline phosphatase; OCN: osteocalcin; PICP: procollagen type I carboxy-terminal propeptide; β-CTX: beta-carboxy-terminal cross-linking telopeptide of type I collagen.

**Supplementary table 2**

**Group Mean %CV in hip and lumbar spine (L2-L4) before, during, and after 45 days of bed rest.**

| %CV | Baseline | BR-45d | R-10d |
|---|---|---|---|
| Mean %CV ± SD in hip | 1.14±0.23 | 0.99±0.12 | 1.11±0.10 |
| Mean %CV ± SD in spine | 1.47±0.14 | 1.52±0.21 | 1.24±0.19 |

All differences are not significant.

**Supplementary figure 1**

Bone mineral density (BMD) of individuals measured by DXA. BMD in hip (A) and lumbar spine (L2-L4) (B) were determined by DXA. Baseline, pre-bed rest; BR-45d, post-45 days of bed rest; R-10d, recovery for 10 days. One-way repeated measures ANOVA was used with post hoc Bonferroni's multiple comparisons. *$P$<0.05, ** $P$<0.01, n=16.

**Supplementary Figure 1**

A

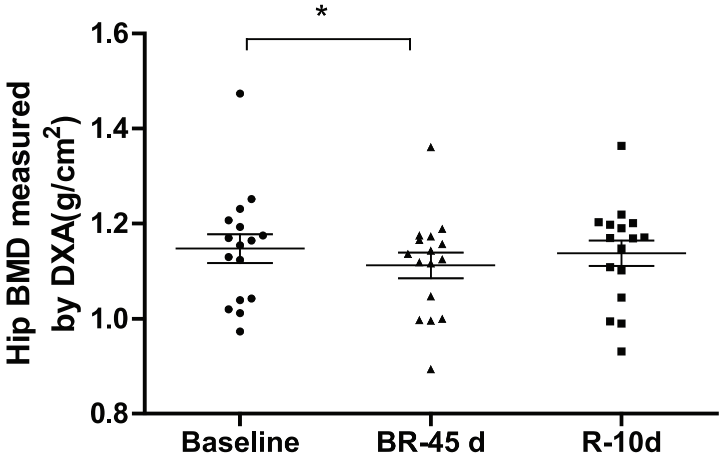


B

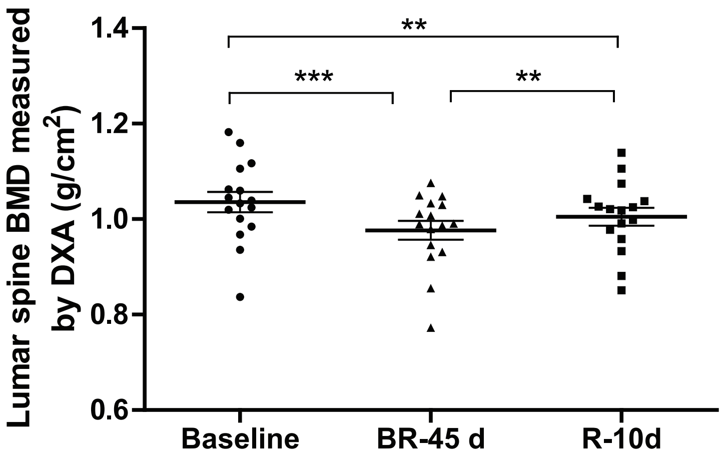

Supplement: Supplementary file 1 [file Presentation1.PDF]
